# Cannabinoid CB1 receptor in dorsal telencephalic glutamatergic neurons drives overconsumption of palatable food and obesity

**DOI:** 10.1038/s41386-021-00957-z

**Published:** 2021-02-08

**Authors:** Inigo Ruiz de Azua, Elena Martin-Garcia, Laura Domingo-Rodriguez, Alejandro Aparisi Rey, Diego Pascual Cuadrado, Larglinda Islami, Petri Turunen, Floortje Remmers, Beat Lutz, Rafael Maldonado

**Affiliations:** 1 Institute of Physiological Chemistry, University Medical Center of the Johannes Gutenberg University Mainz, Mainz, Germany; 2 Leibniz Institute for Resilience Research (LIR), Mainz, Germany; 3 Laboratory of Neuropharmacology-Neurophar, Department of Experimental and Health Sciences, Universitat Pompeu Fabra (UPF), Barcelona, Spain; 4 Microscopy Core Facility, Institute of Molecular Biology (IMB), Mainz, Germany

**Keywords:** Obesity, Reward

## Abstract

Palatable food can promote overfeeding beyond homeostatic requirements, thereby constituting a major risk to obesity. Here, the lack of cannabinoid type 1 receptor (CB1) in dorsal telencephalic glutamatergic neurons (Glu-CB1-KO) abrogated the overconsumption of palatable food and the development of obesity. On low-fat diet, no genotype differences were observed. However, under palatable food conditions, Glu-CB1-KO mice showed decreased body weight and food intake. Notably, Glu-CB1-KO mice were protected from alterations in the reward system after high-fat diet feeding. Interestingly, obese wild-type mice showed a superior olfactory detection as compared to mutant mice, suggesting a link between overconsumption of palatable food and olfactory function. Reconstitution of CB1 expression in olfactory cortex in high-fat diet-fed Glu-CB1-KO mice using viral gene delivery partially reversed the lean phenotype concomitantly with improved odor perception. These findings indicate that CB1 in cortical glutamatergic neurons regulates hedonic feeding, whereby a critical role of the olfactory cortex was uncovered as an underlying mechanism.

## Introduction

Chronic overnutrition beyond energy requirements results in obesity. Obesity facilitates numerous comorbidities, including depression, anxiety, and cognitive deficits [1–4]. Given the prevalence of obesity and its correlation with overconsumption of palatable energy-dense food, understanding the brain circuitry regulating hedonic feeding will be beneficial to tackle the obesity epidemic.

There are two brain systems that control food intake. (i) The homeostatic system adapts feeding and metabolism in response to peripheral signals, acting mainly on hypothalamic and brainstem areas under the control of energy requirements. (ii) The mesolimbic dopamine reward system is involved in the hedonic aspects of feeding, and it is a powerful motivation force to override the homeostatic signals leading to overconsumption of palatable food. Regardless, this is a simplistic view, and there is a functional and anatomical crosstalk between the two regulatory systems.

In this context, endocannabinoids (eCBs) constitute an endogenous orexigenic signal and a key regulator of energy balance through the modulation of cannabinoid type 1 receptor (CB1) activity in central and peripheral tissues [5–10]. Importantly, obesity has been widely associated with an increase in the eCB tone [11]. Thus, blockade of CB1 signaling can reduce body weight, hyperphagia, dyslipidemia, insulin resistance, and other obesity-associated disorders in rodents [12–15] and humans [16]. eCBs play important roles in the positive reinforcing effects of natural rewards such as food, sexual activity, and social interaction [17]. Accordingly, pharmacological [18, 19] and genetic [20] CB1 blockade resulted in a reduction in food reinforcement and motivation in rodents.

The incentive properties of food driving the overconsumption of palatable food are directly related to sensory processing. Thereby, olfaction is one of the major sensory modalities that contribute to the hedonic evaluation of food and food seeking [21]. Therefore, the notion is put forward that changes in olfactory function may modulate food reward. In parallel, the metabolic state of the subject can regulate odor function depending on homeostatic requirements. Therefore, it emerges that olfaction influences appetite behaviors and that the olfactory system is also linked to the metabolic state of the individual [20, 22, 23].

Previous studies showed that CB1 in dorsal telencephalic glutamatergic neurons mediates the orexigenic effect of eCBs in a hunger state [24] by regulating odor detection of food [25], and thereby linking the feeling of hunger to stronger odor processing. Strikingly, mice with deficiency of CB1 in dorsal telencephalic glutamatergic neurons (Glu-CB1-KO) did not show body weight differences as compared to wild-type mice, when mice were fed ad libitum with low-fat diet (LFD) [24]. However, the role of this subpopulation of CB1 in the development of obesity, and in the associated metabolic and behavioral alterations has not yet been clarified. The aim of this study was to investigate whether CB1 in dorsal telencephalic neurons plays a role in feeding behavior in a diet-induced obesity (DIO) model.

## Materials and methods

### Mice

Experiments were performed in male C57BL/6N, Glu-CB1-WT (CB1^floxed/floxed^), and Glu-CB1-KO (CB1^floxed/floxed; heterozygous Nex-Cre^) mice [24]. Mice (2–5 months old) were housed under conditions of controlled temperature (23 ± 1 °C) and illumination (12-h light/dark cycle). Animals were fed with an LFD (13.9 kJ/g: 4.0% fat, 21.1% protein, 56.6% carbohydrate; Altromin, Germany), chocolate diet (16.1 kJ/g: 7.3% fat, 18.6% protein, 61.2% carbohydrate; TestDiet, USA), or high-fat diet (HFD, 21.1 kJ/g: 35.0% fat, 21.3% protein, 29.5% carbohydrate; Altromin, Germany). All experimental procedures were carried out in accordance with the European Directive 2010/63/EU and approved by the Ethical Committee of animal care and use of Rhineland-Palatinate (Germany) and of Barcelona Biomedical Research Park (CEEA-PRBB, agreement N°9687).

### Food intake, body weight monitoring, pair-feeding

Food intake and body weight were measured once a week. For the pair-feeding experiment, Glu-CB1-KO and their Glu-CB1-WT littermates were fed ad libitum on HFD for 4 weeks, and body weight and food intake were recorded  weekly. Starting at fifth week of diet, pair-feeding was performed, whereby Glu-CB1-WT mice received a daily amount of food calculated as the average of daily food intake of the Glu-CB1-KO mice.

### Indirect calorimetry

Energy expenditure was assessed by indirect calorimetry [8]. 10–12-week-old male mice maintained on HFD for 2–3 weeks were individually housed in metabolic chambers (TSE systems, Germany). Following 48 h of acclimation, O_2_ consumption, CO_2_ production, locomotor activity, food and water intake were measured every 15 min for 48 h. Mice had ad libitum access to HFD and water throughout the study.

### Glucose and insulin tolerance test

For the glucose tolerance test, mice that had been fasted overnight (12–16 h) were injected with glucose (2 mg/g i.p.). For insulin tolerance test, fasted (3–5 h) mice were injected with insulin (0.75 IU/kg i.p.; Eli Lilly, Indianapolis, IN). Tail blood glucose levels were measured at defined time intervals (OneTouch® Ultra, LIFESCAN Europe, Switzerland).

### Operant behavioral chambers

Mice were tested during the dark phase of a reverse light cycle. Mouse operant chambers (Model ENV-307A-CT, Med Associates, USA) were used for operant responding maintained by palatable chocolate-flavored pellets (14.4 kJ/g: 20.6% protein, 12.7% fat, 66.7% carbohydrate, TestDiet, USA). After 7 days of exposure to HFD or LFD, all groups were trained under a fixed ratio (FR) 1 schedule of reinforcement in 1-h daily sessions for 5 days, followed by 5 days of training on FR5. The progressive ratio (PR) schedule of reinforcement was used to evaluate the motivation for chocolate-flavored pellets. The response required to earn one single pellet escalated according to the following series: 1, 5, 12, 21, 33, 51, 75, 90, 120, 155, 180, 225, 260, 300, 350, 410, 465, 540, 630, 730, 850, 1000, 1200, 1500, 1800, 2100, 2400, 2700, 3000, 3400, 3800, 4200, 4600, 5000, and 5500. The maximal number of responses that the animal performed to obtain one pellet was the last event completed, referred to as the breaking point (BP). The maximum duration of the PR session was 5 h or until mice did not respond on any lever within 1 h.

### Fluorescence immunohistochemistry (FIHC)

For the detection of the endogenous CB1 and validation of the viral delivery of HA-tagged CB1 transgene, immunostainings were performed against CB1 and HA, respectively. Coronal cryosections (40 μm) were incubated with a rabbit anti-CB1 antibody (1:500; Frontier Institute, Japan, Japan) and monoclonal rat anti‐HA antibody (1:200; Sigma-Aldrich), and were analyzed using the fluorescent Leica DMRA microscope (Leica Microsystems, Germany).

### Confocal imaging

Confocal imaging was performed using Visiscope 5-Elements spinning disk confocal system (Visitron Systems, Germany), built over Nikon TI equipped with Yokogawa CSU-W1 scan head and Prime BSI sCMOS camera (2048 × 2048 pixels, 6.5 µm pixel size, Photometrics). Laser lines of 488 and 561 nm were used for the fluorescence excitation and the fluorescence emission was acquired using filters 525/30 bandpass (Chroma) and 570 long-pass (Chroma) for AF488-rabbit and AF546-rat, respectively. The imaging was performed sequentially to minimize the spectral crosstalk. The large overview of the 30 µm sections were imaged in tile scan mode using a CFI Plan Apo VC ×20/0.75 NA air (Nikon) objective with a pixel binning size of 2 (effective image pixel size of 670 nm). Tile stitching was done using the microscope acquisition software VisiView. The zoomed-in regions were imaged using a CFI Plan Apo VC ×60/1.2 NA water (Nikon) immersion objective (effective image pixel size of 111 nm). The images were prepared using Fiji distribution of ImageJ.

### RNA isolation and real-time PCR analysis

Total RNA was isolated from the olfactory cortex and brain punches. RNA isolation, reverse transcription reaction, and real-time PCR analysis were described previously [26]. In order to evaluate the rescue of CB1 gene expression after AAV-mediated gene delivery, real-time PCR analysis was carried out with Taqman mouse CB1 primers (Mm00432621_s1), and we also designed a specific pair of primers hydridizing to mouse CB1 and rat CB1 transgene (forward: TTC AAA CTG GGT GGG GTT AC; reverse: GCC TGG TGA CGA TCC TCT TA). The latter primers were used in combination with PowerUp SYBR Green Master Mix (Life Technologies, Germany).

### Synaptosomal preparation

Synaptosomal preparation was carried out as described previously [27]. In brief, isolated olfactory bulb (OB) was homogenized in a glass grinder in 320 mM glucose TVEP buffer (10 mM Tris-HCl pH = 7.4, 5 M NaF, 1 mM Na_3_VO_4_, 1 mM EDTA, 1 mM EGTA). The homogenate was centrifuged (10 min, 1000 × *g* at 4 °C). Supernatant was collected, and an aliquot was stored at −80 °C as a total fraction, before isolation of synaptosomes. After thawing, the supernatant was centrifuged (15 min, 15,000 × *g* at 4 °C) in order to obtain crude synaptosomes. Then, the pellet was re-suspended in 35.6 mM glucose TVEP buffer and placed on ice for 30 min. This step led to hypoosmotic lysis of the synaptosomes to release synaptic vesicles and other cytoplasmic organelles. Finally, the resuspension was centrifuged (20 min, 20,000 × *g* at 4 °C), yielding the synaptosomal fraction in the pellet.

### Western blot

Olfactory cortex lysates in RIPA buffer (50 mM Tris-HCl pH = 7.4, 150 mM NaCl, 1 mM EDTA, 1% NP-40; 5% Na-deoxycholate) were centrifuged (10,000 r.p.m., 10 min at 4 °C), and supernatants were collected. Samples were run on a 10% polyacrylamide gel, transferred to nitrocellulose membranes, and incubated with primary antibodies for CB1 (1:1000; Immunogene), synaptotagmine (1:500, sc136089; Santa Cruz), and β-actin (1:1000, ab1801; Abcam). The secondary antibody (HRP-conjugated anti-mouse and anti-rabbit antibody; Jackson ImmunoResearch) was visualized using the ECL detection system (GE Healthcare Life Sciences). Quantification of the bands was done with Fusion and Bio‐1D software (Vilber Lourmat, Germany). Finally, the ratio between the signals of CB1 and loading control (β-actin) was calculated for each sample.

### Olfactory habituation

A novel neutral odor (butter-vanillin dissolved in 50% mineral oil; Sigma-Aldrich) was used during the test; and mineral oil as control. Food was removed 2 h prior to the test. For odor presentations, 10 μl of each solution were deposited onto a cotton swab and then placed inside a holder. The task consisted of 3-min odor presentations separated by 5-min inter-trial intervals. In a given session, mice were first habituated to the new conditions for 30 min including the swab. Later, mice were presented with the swab containing the mineral oil only. They were then presented with the odorant during two successive trials. Exploration of the odor source was defined as directing the nose at a distance <1 cm from the tip of the swab, and/or touching it with the nose.

### Buried food-seeking test

First, mice were habituated to the chocolate pellets. The food was removed 2 h prior to the test. Mice were habituated for 5 min to the test cage with new bedding. Mice were then put back to the home cage. Meantime, chocolate pellets were buried 2 cm beneath of the bedding surface in one of the corners of the test cage. Mice were put back into the test cage in the opposite corner in regard to the pellet. Time was recorded until mice retrieved the chocolate pellet.

### Re-expression of CB1 in olfactory cortex of Glu-CB1-KO mice

Adult male mice were injected bilaterally with AAV-stop-HA-rCB1 [28] via stereotaxic surgery into the medial (+2.6 mm AP, ±0.5 mm ML, −4.0 mm DV relative to bregma) and lateral (2.3 mm AP, ±1.5 mm ML, −4.2 mm DV relative to bregma) anterior olfactory nucleus (AON) of Glu-CB1-KO and Glu-CB1-WT mice.

### Statistics

Results are expressed as mean ± SEM. Data were analyzed by unpaired two-tailed Student’s *t*-test or by analysis of variance (ANOVA) followed by appropriate post hoc tests. Data with repeated measurements were analyzed by repeated measures ANOVA followed by post hoc tests. Energy expenditure (in kcal/h) and food intake (in g per week) were analyzed by ANCOVA using body weight as covariate. The behavioral data of operant responding during FR1 and FR5 were analyzed using three-way repeated measures ANOVA. Data of BP were analyzed using two-way ANOVA with diet and genotype as a between-subjects factor in the PR tests. The statistical analysis was performed using GraphPad Prism 5.0 software and SPSS v15.0 (SPSS Inc., USA). Data with *p* < 0.05 were considered as statistically significant. Supplementary Table 2 includes detailed information of statistical analysis performed in this study.

## Results

### Glu-CB1-KO mice are protected against DIO

**Fig. 1:**
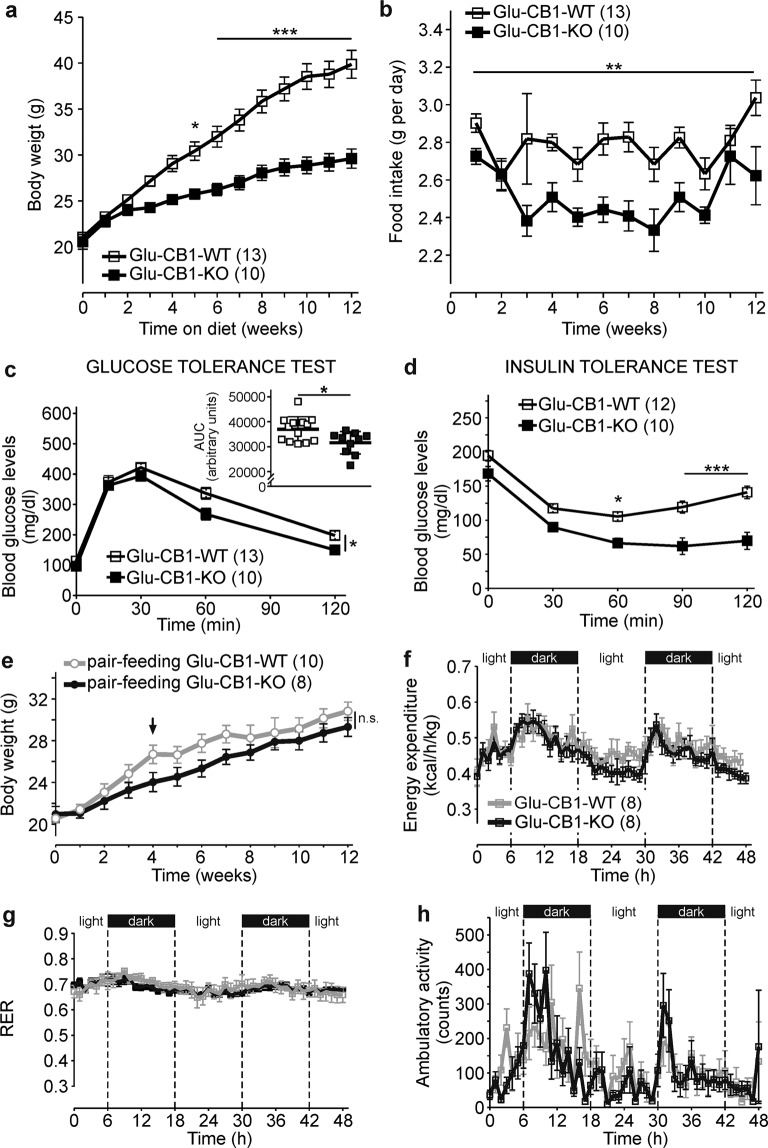
CB1 deletion in dorsal telencephalic glutamatergic neurons is sufficient to protect against diet-induced obesity and impaired glucose homeostasis. **a** Body weight growth curves and **b** food intake of Glu-CB1-KO (*n* = 10) and Glu-CB1-WT (*n* = 13) mice on high-fat diet (HFD). **c** Glucose tolerance test (area under the curve (AUC) for each mouse is represented in the inset), and **d** insulin tolerance test in Glu-CB1-KO (*n* = 10) and Glu-CB1-WT (*n* = 13–12) mice fed on HFD treatment. **e** HFD pair-feeding experiment starting after 4 weeks of HFD in Glu-CB1-KO (*n* = 8) and Glu-CB1-WT (*n* = 10) mice. **f** Energy expenditure of Glu-CB1-KO (*n* = 8) and Glu-CB1-WT (*n* = 8) performed at second–third week of HFD treatment. **g** Respiratory exchange ratio (RER) and **h** ambulatory activity during indirect calorimetry assays of Glu-CB1-KO (*n* = 8) and Glu-CB1-WT (*n* = 8). Repeated measures two-way ANOVA analysis and Bonferroni’s multiple comparison tests at indicated time points, *p < 0.05; **p < 0.01; ***p < 0.001. Food intake was also analyzed by ANCOVA analysis using body weight as covariate, ***p < 0.001. AUC was analyzed with an unpaired two-tailed Student’s *t*-test. *p < 0.05 Glu-CB1-WT vs Glu-CB1-KO. Data are shown as mean ± SEM.

Glu-CB1-KO mice were challenged in the DIO model. Strikingly, the lack of CB1 in this neuronal population was sufficient to protect against weight gain compared with wild-type control mice (Glu-CB1-WT) (Fig. 1a). The lower weight gain was associated with a decreased food intake (Fig. 1b), even when the effect of body weight was controlled after ANCOVA analysis (genotype, *F*_(1,272)_ = 25.996, *p* < 0.001). As expected, lean HFD-fed Glu-CB1-KO mice were protected from glucose intolerance and insulin resistance (Fig. 1c, d). In the pair-feeding experiment, Glu-CB1-WT mice were fed with the same amount of food that was daily consumed by Glu-CB1-KO mice, finally blunting the body weight difference between genotypes (Fig. 1e). Furthermore, energy expenditure (EE), respiratory exchange ratio (RER), and locomotor activity were evaluated during the second and third week of HFD before body weight differences appeared (Fig. 1f–h). ANCOVA analysis of EE did not show statistically significant differences between both genotypes when body weight was used as a covariate (Fig. S1), excluding body weight as a confounding factor [29]. There were no significant genotype differences regarding energy dissipation and physical activity (Fig. 1f–h).

### Glu-CB1-KO show reduced overconsumption of palatable food

**Fig. 2:**
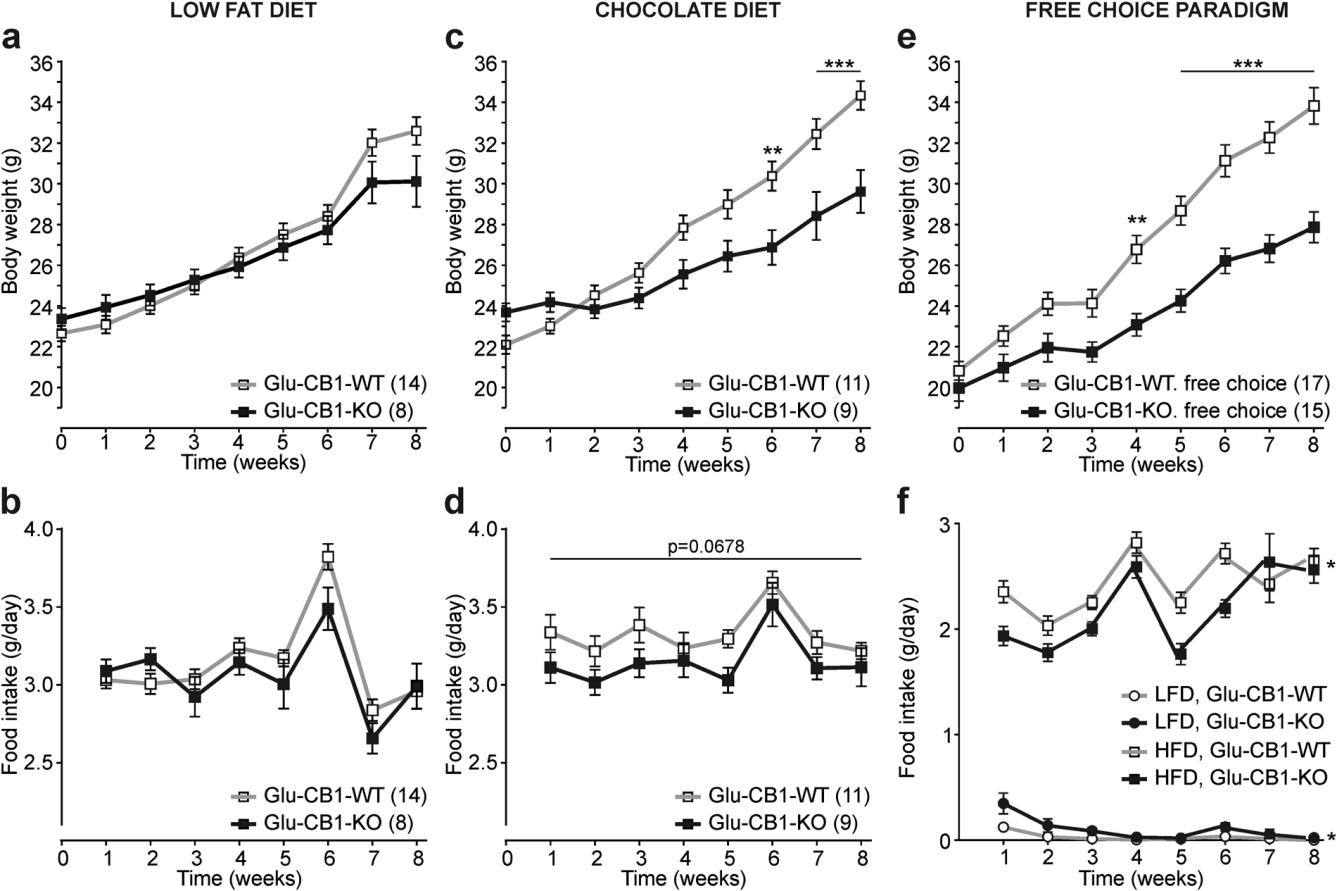
Glu-CB1-KO mice have a reduced overconsumption only of palatable food. **a**, **b** Body weight growth curves (**a**) and food intake (in grams per day) (**b**) of Glu-CB1-KO (*n* = 8) and Glu-CB1-WT (*n* = 14) mice under low-fat diet (LFD). **c**-**d** Chocolate-flavored pellets have the same caloric content but higher sucrose concentration than LFD. Body weight (**c**) and food intake (**d**) Glu-CB1-KO (*n* = 9) and Glu-CB1-WT (*n* = 11) mice fed with chocolate-flavored pellets. **e**, **f** In the free-choice paradigm, all mice had free access to LFD and HFD. Body weight (**e**) and food intake of LFD and HFD (**f**) of Glu-CB1-KO (*n* = 15) and Glu-CB1-WT (*n* = 17) mice. Repeated measures two-way ANOVA analysis and Bonferroni’s multiple comparison tests at indicated time points, *p < 0.05; **p < 0.01; ***p < 0.001 Glu-CB1-WT vs Glu-CB1-KO. Food intake was analyzed by ANCOVA analysis using body weight as covariate, **p < 0.01 (**d**); *p < 0.05 (HFD, **f**). Data are shown as mean ± SEM.

Glu-CB1-KO mice did not show body weight and food intake differences on an LFD, confirming the previous study [24] (Fig. 2a, b), giving the basis to investigate the consequences of different dietary regimens using palatable food. First, we fed mice with isocaloric chocolate-flavored food. As shown in Fig. 2c, d, we found a decreased weight gain and food intake in Glu-CB1-KO, when food intake difference was controlled for body weight by analysis of the covariance (chocolate diet: genotype, *F*_(1,157)_ = 8.575, *p* < 0.01). Furthermore, we challenged these mice in a free-choice paradigm where mice had ad libitum access to both LFD and HFD. Both genotypes showed a preference for HFD (Fig. 2f), but importantly, Glu-CB1-WT mice had a significantly higher initial (during the first week) preference for the palatable diet than Glu-CB1-KO mice (Fig. S2). Consequently, Glu-CB1-WT mice consumed significantly more HFD compared with Glu-CB1-KO, and thereby they had a higher weight gain (Fig. 2e, f). Differences in food intake were also observed after analysis of the covariance using body weight as a covariate (free choice: genotype, *F*_(1,226)_ = 5.269, *p* < 0.05). Notably, the lowered caloric intake and body weight in Glu-CB1-KO mice are only observed with palatable food but not with LFD, suggesting a dysfunction in food reinforcement behavior in the mutant mice.

### Increased motivation to obtain palatable food after HFD withdrawal is blunted in Glu-CB1-KO mice

**Fig. 3:**
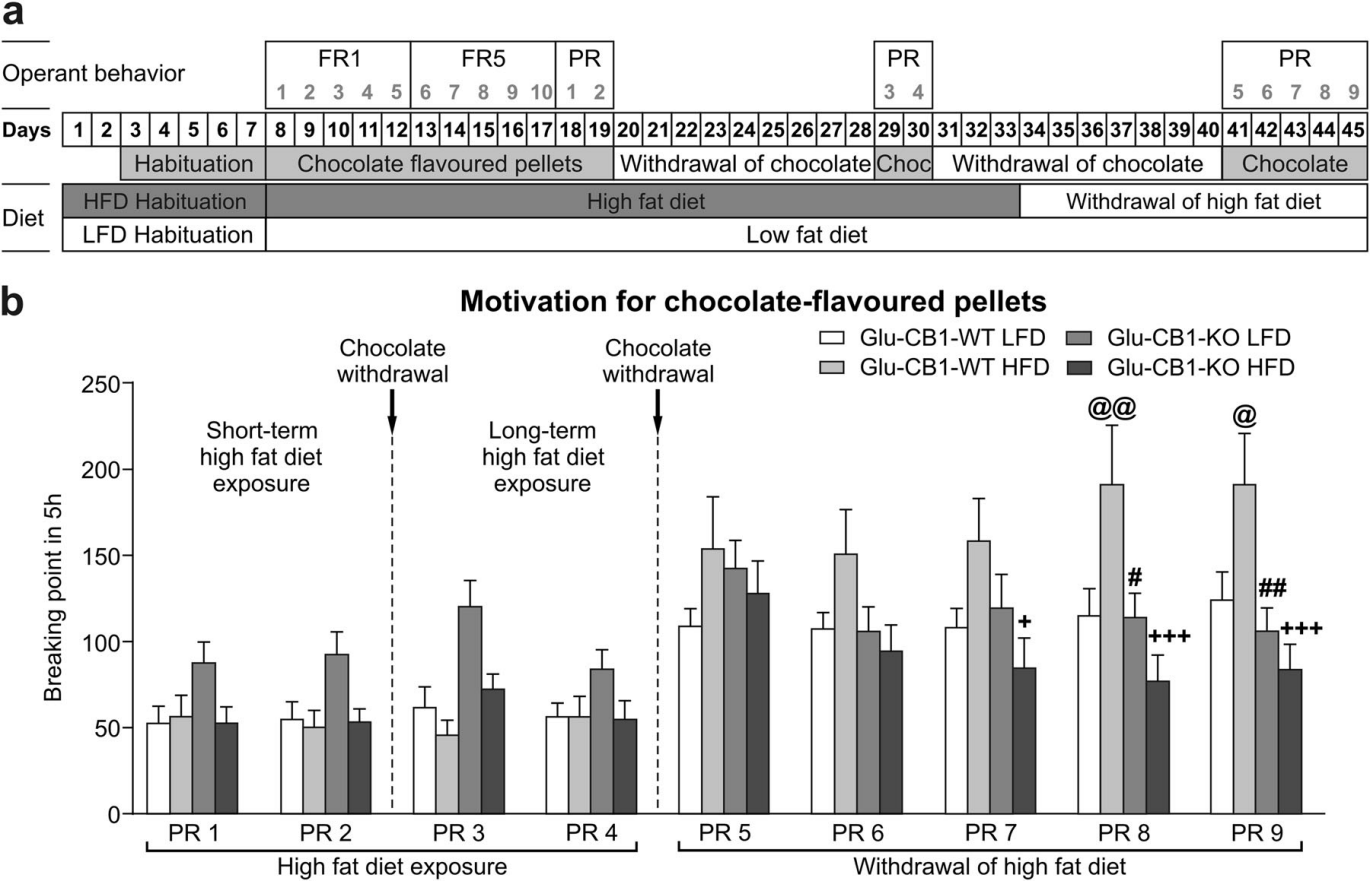
Increased motivation to obtain palatable food after HFD withdrawal is blunted in Glu-CB1-KO mice. **a** Experimental sequence to study motivation for chocolate-flavored pellets in Glu-CB1-WT and Glu-CB1-KO mice exposed to LFD and HFD. Mice were habituated to LFD or HFD and to chocolate-flavored pellets 1 week before the start of the operant behavior. Then, mice were trained for operant conditioning maintained by chocolate-flavored pellets under a fixed ratio (FR) 1 schedule of reinforcement on 1-h daily sessions during 5 days followed by 5 days of FR5 schedule of reinforcement paired with the presentation of a cue-light. After FR training, mice were exposed to two PR sessions followed by 9 days of chocolate-flavored pellets withdrawal and to two PR sessions more. After a period of 7–10 days of HFD and chocolate withdrawal, mice were tested again in the PR schedule during five consecutive days. **b** Motivation for chocolate-flavored pellets during high-fat diet exposure and high-fat diet withdrawal. Breaking point achieved in each 5 h progressive ratio session. Newman–Keuls post hoc test following two-way ANOVA, ^@^*p* < 0.05, ^@@^*p* < 0.01, LFD/Glu-CB1-WT vs HFD/Glu-CB1-WT; ^#^*p* < 0.05, ^##^*p* < 0.01, HFD/Glu-CB1-WT vs LFD /Glu-CB1-KO; ^+^*p* < 0.05, ^+++^*p* < 0.001, HF/Glu-CB1-WT vs HFD/Glu-CB1-KO. Data are expressed as mean ± SEM; 15–26 mice per group.

Next, we decided to study the motivation of these mice for palatable food using a self-administration paradigm (Fig. 3a). During the operant behavior, Glu-CB1-KO mice obtained a slightly increased number of reinforcers in the first FR1 session compared with Glu-CB1-WT mice (Fig. S3a-b), suggesting a superior learning. HFD-fed Glu-CB1-WT mice showed a significantly higher body weight and food intake than HFD-fed mutant mice (Fig. S3c, d) as observed above. Repeated measures ANOVA revealed that the interaction between genotype and time was significant during FR1, indicating that Glu-CB1-WT mice increased the number of pellets obtained during sessions, whereas mutants do not and remain stable during the whole period. We evaluated the motivation using a PR schedule. As it is indicated by the  breaking point, no significant differences in motivation to obtain chocolate-flavored pellets (PR1 and PR2) were revealed among the different groups after a short-term exposure to LFD or HFD (Fig. 3b). Furthermore, we evaluated the effect of a chocolate withdrawal period on the operant behavior in these mice (PR3 and PR4), where no significant effects were found (Fig. 3b). Next, we evaluated the behavioral changes following the removal of HFD. After 1 week of HFD withdrawal, Glu-CB1-WT mice showed a higher motivation for chocolate-flavored pellets than the other three groups during five consecutive days, although significant changes appeared only during the last two sessions (Fig. 3b). Notably, the absence of CB1 in dorsal telencephalic glutamatergic neurons was sufficient to protect the mice against the behavioral sensitization in food motivation triggered by the HFD withdrawal (Fig. 3b).

### Expression of CB1 in olfactory system

Based on above findings, we analyzed the CB1 expression in corticolimbic areas that are part of the reward system by FIHC in these mice on both LFD and HFD. Glu-CB1-KO mice on HFD showed a strong reduction of CB1 protein levels in the OB. Confocal imaging of FIHC revealed that CB1 levels are strongly decreased in the granule cell layer (GCL) of olfactory bulb (OB), yet not a complete loss was observed (Fig. S4a). GCL cells are mainly inhibitory GABAergic neurons that receive glutamatergic inputs from olfactory cortical areas, such as AON and piriform cortex. The decreased CB1 protein levels in Glu-CB1-KO mice were confirmed by western blot analysis in isolated OB (Fig. S4b). The analysis of CB1 mRNA levels by qPCR indicated a significant decrease in isolated AON fractions from Glu-CB1 KO mice compared with Glu-CB1-WT littermates on HFD (Fig. S4c, mean diff. −0.845, 95% CI −1.22 to −0.471, *p* < 0.001). Additionally, we further investigated the presynaptic CB1 location by western blot analysis of total and synaptosomal fractions of the OB in these mice, showing approx. fourfold decreased expression levels in both fractions in Glu-CB1-KO mice as compared to Glu-CB1-WT mice (Fig. S4d, genotype, *F*_(1,12)_ = 15.55, *p* < 0.01). Therefore, we concluded that the reduced CB1 expression in the OB of the Glu-CB1-KO mice is due to the lack of presynaptic CB1 in the excitatory inputs from olfactory cortical areas.

### Glu-CB1-KO mice exhibit reduced odor perception and food-seeking behavior

**Fig. 4:**
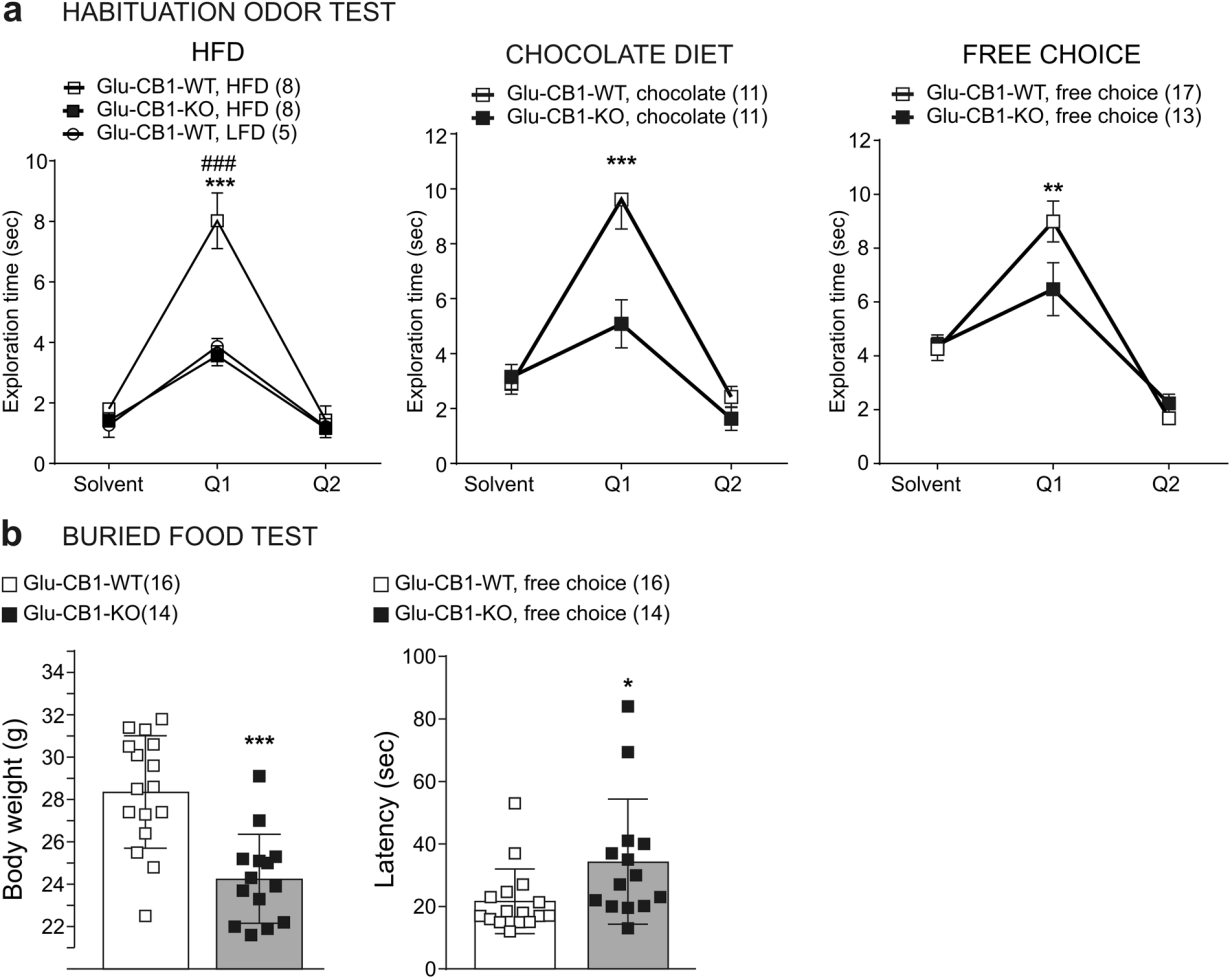
Glu-CB1-KO mice exhibited reduced odor perception and food-seeking behavior. **a** Habituation odor test in Glu-CB1-WT and Glu-CB1-KO mice fed with different diet regimens: LFD or HFD; chocolate-flavored pellets; free-choice paradigm. Q1 refers to the first exposure to the odor and Q2 refers to the second exposure. Bonferroni post hoc test following two-way ANOVA, ^###^*p* < 0.001 LFD/Glu-CB1-WT vs HFD/Glu-CB1-WT; **p < 0.01, ***p < 0.001 Glu-CB1-WT vs Glu-CB1-KO. **b** Buried food test, body weight, and the latency to find the chocolate pellet in Glu-CB1-WT (*n* = 16) and Glu-CB1-KO (*n* = 14) mice fed using the free-choice paradigm. Unpaired two-tailed Student’s *t*-test, *p < 0.05, ***p < 0.001 free choice/Glu-CB1-KO vs free choice/Glu-CB1-WT. Data are expressed as mean ± SEM.

Given the expression of CB1 in the olfactory system and its role in olfactory perception [25], we tested odor behavior in these mice on the different diets. We performed a habituation odor test using one novel odor. In LFD-fed mice, we did not find significant differences in the exploration time at the first exposure to the novel odor (Q1) or in odor habituation (Q2) between genotypes (Fig. S5a) or after short exposure to HFD (Fig. S5b). However, once body weight changes emerged, obese HFD-fed Glu-CB1-WT mice showed an enhanced exploration compared to lean HFD-fed Glu-CB1-KO, but exclusively in the first exposure to the odor (Q1) (Fig. 4a), indicating an increased odor detection without changes in odor habituation (Q2). The increased odor perception in Glu-CB1-WT mice was also found in mice on chocolate and free-choice diets once body weight changes were present (Fig. 4a). Notably, Glu-CB1-WT mice on HFD showed a higher response than Glu-CB1-WT mice on LFD (Fig. 4a). The metabolic status of the mice strongly affected the responses in the habituation odor test as suggested by a significantly positive correlation between body weight and exploration time in Glu-CB1-WT mice (Fig. S5c, *r*_(33)_ = 0.508, *p* = 0.002). Interestingly, LFD-fed Glu-CB1-WT mice showed a better odor perception than HFD-fed mice in fasting conditions (Fig. S5d), while the differences between HFD-fed Glu-CB1-WT and Glu-CB1-KO mice were blunted under these conditions.

We also evaluated HFD-fed Glu-CB1-KO and Glu-CB1-WT mice in the buried food-seeking test using chocolate-flavored pellets as an odor cue. We observed that the latency to find the food was significantly longer in HFD-fed lean Glu-CB1-KO than in obese Glu-CB1-WT mice, indicating potential olfactory deficits and/or decreased motivation for chocolate-flavored pellets (Fig. 4b, *t*_(28)_ = 0.224, *p* = 0.034). The same phenotype was found in LFD-fed mice (Fig. S5e). Overall, the lack of CB1 in dorsal telencephalic neurons decreased olfactory discrimination between background odors and the detection of food cues.

### Rescued CB1 expression in the olfactory cortex of Glu-CB1-KO mice partly restores DIO

**Fig. 5:**
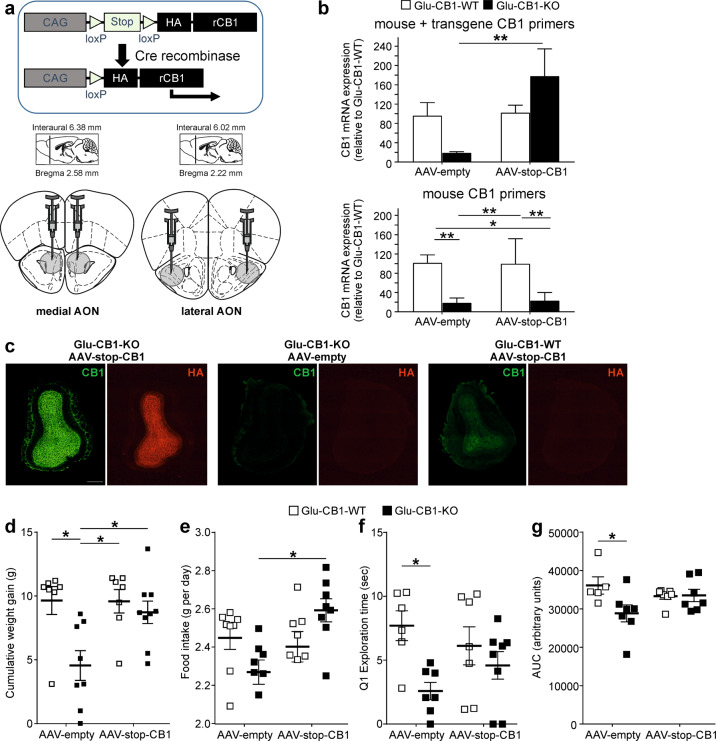
Rescued CB1 expression in glutamatergic neurons in the olfactory cortex partly restored diet-induced obesity in Glu-CB1-KO mice. **a** Scheme of adeno-associated viral bilateral injections in the medial and lateral AON for the rescue of CB1 in olfactory cortical areas in Glu-CB1-KO mice. **b** qPCR analysis of CB1 gene expression in brain punches of the AON in the four AAV-injected groups of mice. Use of different primer pairs allowed distinction of total CB1 expression (endogenous mouse and transgenic CB1; upper panel) and only mouse CB1 expression (lower panel). **c** Representative immunofluorescence images of CB1 and HA-tag protein expression in the OB of AAV-stop-CB1 Glu-CB1-KO, AAV-empty Glu-CB1-KO mice, and AAV-stop-CB1 Glu-CB1-WT mice. Scale bar: 400 μm. Cumulative weight gain (**d**) and food intake (**e**) of AAV-injected Glu-CB1-WT and Glu-CB-KO mice during the last 8 weeks after AAV-mediated gene delivery. **f** Exploration time to first exposure to the odor (Q1) during the habituation odor test and **g** area under the curve (AUC) of glucose tolerance test in the same experimental groups of mice. Sidak multiple comparison test following two-way ANOVA: *p < 0.05, **p < 0.01. Data are expressed as mean ± SEM.

As in Glu-CB1-KO mice CB1 is deleted in a large population of glutamatergic neurons in cortical areas including the olfactory system, we asked whether the reconstitution of CB1 expression specifically in the olfactory cortex is able to reverse the lean phenotype under HFD treatment and the reduced odor-guided behavior. To this end, we re-expressed CB1 in glutamatergic neurons of cortical olfactory areas using an adeno-associated virus (AAV) approach (Fig. 5a). We bilaterally injected an AAV vector carrying a Cre-dependent HA-tagged CB1 transgene (AAV-stop-HA-CB1) or control (AAV-stop-empty) into the medial and lateral AON of Glu-CB1-KO and -WT mice in the fourth week of HFD treatment. We verified HA-CB1 expression both in olfactory cortical areas and OB of AAV-stop-HA-CB1-injected Glu-CB1-KO mice on week 4 after injection (Figs. 5b, c and S6). Eight weeks after AAV injections, the selective rescue of CB1 in cortical olfactory areas of Glu-CB1-KO mice blunted the differences in cumulative weight gain when compared with both Glu-CB1-WT groups (AAV-stop-empty and AAV-stop-HA-CB1), whereas AAV-stop-empty Glu-CB1-KO mice stayed lean (Fig. 5d and S7a). Accordingly, AAV-stop-CB1-HA injected Glu-CB1-KO mice showed a significantly higher cumulative weight gain than AAV-stop-empty Glu-CB1-KO mice (Fig. 5d). Two-way ANOVA analysis showed a significant interaction effect between genotype and AAV (*F*_(1,27)_ = 4.262, *p* = 0.049). The increased weight gain in AAV-stop-CB1-HA Glu-CB1-KO mice was associated with an increased food intake (Fig. 5e and S7b). These mice also had a partially rescued Q1-odor response during habituation odor test (Fig. 5f and S7c) and a partially impaired glucose homeostasis during glucose tolerance test (Fig. 5g and S7d). Two-way ANOVA analysis showed a significant interaction effect between genotype and AAV in cumulative weight gain (*F*_(1,27)_ = 4.262, *p* = 0.049), food intake (*F*_(1,28)_ = 7.684, *p* = 0.001), and AUC (*F*_(1,22)_ = 4.466, *p* = 0.046) substantiating the critical role of olfactory CB1 in overfeeding mediated obesity.

## Discussion

Here, we provide new insights in understanding the role of the eCB system in the regulation of overconsumption of palatable food leading to obesity. These findings indicate the specific role of CB1 in dorsal telencephalic glutamatergic neurons in hedonic feeding and, by extension, in overfeeding-induced obesity. The underlying mechanism involved in overconsumption of palatable food is partly mediated by the expression of CB1 in olfactory cortical areas controlling food seeking and feeding behavior in obese mice.

When we challenged Glu-CB1-KO mice in a DIO model, we found that the lack of CB1 in this subset of neurons protects against weight gain, overfeeding, and glucose and insulin intolerance. Importantly, we could not find changes in EE in these mice before body weight changes emerged. Pair-feeding was sufficient to fully blunt body weight differences between both genotypes. These findings underline that the protective role against HFD in Glu-CB1-KO mice was primarily mediated by a reduced food consumption. Interestingly, the deletion of CB1 in forebrain principle projecting neurons and sympathetic neurons elicited a similar lean phenotype, but these mutant mice showed an enhanced sympathetic tone leading to increased lipid oxidation and brown adipose tissue (BAT) thermogenesis, but not changes in food intake [5]. In contrast, CB1 expression in dorsal telencephalic neurons mediates the orexigenic effects of eCBs when mice were fasted 24 h [24, 25]. These findings reveal that CB1 expression in different neuronal populations promotes energy store and obesity development through complementary mechanisms.

Using two other palatable diets (isocaloric chocolate diet and free-choice) corroborated the reduced overconsumption of palatable food in Glu-CB1-KO mice. When mice could choose between LFD and HFD, both genotypes had a strong preference for HFD, although the initial preference for HFD was less in the Glu-CB1-KO mice and they consumed less palatable diet than Glu-CB1-WT mice. The lowered initial preference for the palatable diet might be a protective factor in the development of obesity caused by reduced reinforcing properties of the palatable food in these animals. Importantly, the control of feeding by CB1 expression in these neurons seems to be independent of the homeostatic mechanisms as suggested by the lack of phenotype in LFD-fed mice, confirming previous studies [24]. Overall, these subtle but continuous differences in hedonic feeding are sufficient to reduce the overconsumption of the palatable diet and protect the mice against obesity in an obesogenic environment. Our current findings revealed that Glu-CB1-KO mice have a reduced hedonic feeding which might be related to changes in the pleasure of eating or in food reinforcement.

With this in mind, we measured the food motivation in these mice in a PR using chocolate-flavored pellets as reinforcers. First, we could not observe a correlation between hyperphagia and increased motivation for palatable food in HFD-fed Glu-CB1-WT mice. This is most likely because the willingness to work for the chocolate pellets is reduced when mice have ad libitum access to hypercaloric food in the home cage [30]. Moreover, palatable diet is still reinforcing in the Glu-CB1-KO mice as shown in the free-choice study. Therefore, we examine the behavior upon removal of the HFD. After HFD withdrawal, HFD-fed Glu-CB1-WT mice displayed a significantly higher BP, which was indicated by an increased amount of effort that the mice were willing to perform for food reward. Importantly, this effect was not observed in Glu-CB1-KO mice on HFD, suggesting a protective role of the deletion of CB1 in sensitization to the reward after long-term HFD exposure and subsequent withdrawal. HFD removal was reported to increase operant responding to sucrose and food rewards in obese animals [30, 31], but not in obesity-resistant rats [30] and when mice were fed with LFD [30]. Extended access to cafeteria diet in rats increased the brain electrical stimulation in the lateral hypothalamus, pointing out a deficit in the brain reward function [32]. Therefore, both our data and previous studies [26, 30, 32] suggest that the development of obesity is coupled with neuronal adaptations in the reward system that drive the overeating in order to alleviate the diminished reward, in which CB1 in dorsal telencephalic neurons occupies a key regulatory function. Supporting these findings, we have recently showed that the lack of CB1 in this neuronal subpopulation did not fully mitigate the development of food addiction but significantly promoted a resilient phenotype [33].

Subsequently, we investigated the underlying mechanism of the overconsumption in HFD-fed Glu-CB1-WT mice and the protective role of CB1 signaling blockade in cortical excitatory neurons. In Glu-CB1-KO mice, deletion of CB1 occurred in glutamatergic neurons in neocortex, hippocampus, cortical regions of amygdala, and subcortical regions containing cortical glutamatergic projections [34, 35]. Some of these brain areas are part of the reward system, thereby we cannot exclude a potential contribution of the CB1 loss in other brain areas involved in hedonic feeding, such as striatum, amygdala, or hypothalamic areas [35]. In dorsal telencephalon, CB1 is highly expressed in GABAergic interneurons [34]. Interestingly, we found a strong reduction of the CB1 expression in the GCL of the OB of Glu-CB1-KO mice as revealed previously [25]. GCL contains mainly inhibitory GABAergic neurons that receive glutamatergic inputs from olfactory cortical areas [36]. In agreement with this, we found significantly reduced CB1 mRNA levels in the olfactory cortex of Glu-CB1-KO mice.

In mammals, the sense of smell is widely involved in eating behaviors from food detection to consumption. However, the impact of obesity in odor perception still remains elusive. During the habituation odor test, obese Glu-CB1-WT mice fed with palatable food showed an increased exploration time at the first exposure to a novel odor compared with Glu-CB1-KO mice on the same diet. The enhanced odor detection in obese mice was also corroborated in the buried food test, where a reduced latency to find the buried chocolate pellet was observed. Consistent with this finding, obese ob/ob mutant mice lacking leptin performed significantly better than WT animals in a food-finding test [37]. DIO-induced elevated eCB tone might explain the increased odor detection in HFD-fed Glu-CB1-WT mice as compared with LFD-fed Glu-CB1-WT mice. Overall, our data show that the lack of CB1 in dorsal telencephalic neurons prevented the obesity-induced increase in odor behavioral response including food seeking.

Since odor detection guides behaviors including appetitive and feeding behaviors [37–39], we hypothesized that the lack of CB1 in the excitatory neurons of olfactory cortical areas plays a major role in the overfeeding of palatable food. Indeed, the loss of olfaction in healthy individuals is associated with a reduced enjoyment of food [40]. In order to validate our hypothesis, we re-expressed CB1 in the excitatory neurons of the olfactory cortex in Glu-CB1-KO mice using a Cre-dependent viral delivery approach. Importantly, the re-expression of CB1 enabled promoting an obese phenotype in Glu-CB1-KO mice as compared with control operated mutant mice. In Glu-CB1-KO mice, the increased weight gain was associated with an increased food intake. Furthermore, there was a partial improvement in olfactory detection after re-expression of CB1 in Glu-CB1-KO mice during the habituation odor test as compared with AAV-stop-empty injected Glu-CB1-KO mice. However, it is still unclear if such changes in odor processing contribute to obesity development or whether it is only a consequence of increased body weight.

Gender differences have been reported in obesity and reward-seeking behavior with a higher prevalence in women [41, 42]. The greater vulnerability in women could be related to intrinsic biological differences between female and male subjects [43, 44], apart from environmental and cultural factors. Further studies will clarify whether the role of CB1 in olfactory cortex regulating hedonic feeding differs between female and male mice.

In conclusion, our data indicate the critical role of CB1 expression in dorsal telencephalic excitatory neurons in regulating hedonic feeding behavior and the development of obesity. The deletion of CB1 in these neurons protects against pathological effects of DIO by decreasing feeding behavior of the palatable food, including obesity-associated neuronal adaptations in the mesolimbic dopamine reward system. Strikingly, the reduced caloric intake and body weight was observed when mice were fed with palatable food, but not in LFD. These mutant mice also showed a decrease of CB1 expression in GCL of the OB, which contributes to a decrease in exploratory food odor-guided behavior after long-term HFD exposure. Notably, the reconstitution of CB1 locally in olfactory cortical areas in Glu-CB1-KO mice using AAV gene delivery partially reversed the phenotype of Glu-CB1-KO mice on HFD, facilitated weight gain, increased food intake, and enhanced odor detection. These findings show that CB1 expression in cortical glutamatergic neurons is particularly relevant to promote overconsumption of palatable food and, by extension, obesity. The uncovered new role of CB1 in the olfactory system seem to be crucial for the emergence of DIO.

## Funding and disclosure

We acknowledge the grants from the German Research Foundation DFG to B.L. (CRC1193) and A.A.R. and B.L. (CRC1080) and the Boehringer Ingelheim Foundation to B.L. and I.R.A. We thank the financial support of the Spanish “Ministerio de Ciencia, Innovación y Universidades” (#AEI-SAF2017-84060-R FEDER), the “Instituto de Salud Carlos III” (#RD16/0017/0020), the “Plan Nacional Sobre Drogas” of the Spanish “Ministerio de Sanidad, Servicios Sociales e Igualdad” and the AGAUR of the “Generalitat de Catalunya” (#2017-SGR-669 and #ICREA-Acadèmia 2015) to R.M. and Fundació La Marató-TV3 (#2016/20-30) and Plan Nacional Sobre Drogas (#PNSD-2019I006) to E.M.-G. The authors declare no conflict of interest. Open Access funding enabled and organized by Projekt DEAL.

## Supplementary information

Supplementary information

Supplementary Table-1

Supplementary Table-2

Figure S1

Figure S2

Figure S3

Figure S4

Figure S5

Figure S6

Figure S7
