# Supplementary material for: Cannabinoid CB1 receptor in dorsal telencephalic glutamatergic neurons drives overconsumption of palatable food and obesity
Source: Neuropsychopharmacology. 2021 Feb 8;46(5):982–91. doi: 10.1038/s41386-021-00957-z (PMC8105345; doi:10.1038/s41386-021-00957-z)
Supplement: Supplementary file 2 — Supplementary Table-1 [file 41386_2021_957_MOESM2_ESM.docx]

**Supplementary Table S1: Discrimination between active and inactive nose-pokes during FR1 and FR5**

|  | | | |
| --- | --- | --- | --- |
| **Group** | **Mean** | **Standard error** | **N** |
|  |  |  |  |
|  | FR1 |  |  |
| Glu-CB1-WT LFD | 74.278 | 2.870 | 15 |
| Glu-CB1-WT HFD | 73.287 | 2.695 | 17 |
| Glu-CB1-KO LFD | 78.575 | 2.180 | 26 |
| Glu-CB1-KO HFD | 71.466 | 2.425 | 21 |
|  |  |  |  |
|  | FR5 |  |  |
| Glu-CB1-WT LFD | 81.218 | 2.538 | 15 |
| Glu-CB1-WT HFD | 85.858 | 2.384 | 17 |
| Glu-CB1-KO LFD | 86.099 | 1.928 | 26 |
| Glu-CB1-KO HFD | 81.562 | 2.145 | 21 |
