# Supplementary material for: Cannabinoid CB1 receptor in dorsal telencephalic glutamatergic neurons drives overconsumption of palatable food and obesity
Source: Neuropsychopharmacology. 2021 Feb 8;46(5):982–91. doi: 10.1038/s41386-021-00957-z (PMC8105345; doi:10.1038/s41386-021-00957-z)
Supplement: Supplementary file 3 — Supplementary Table-2 [file 41386_2021_957_MOESM3_ESM.docx]

**Supplementary Table 2. Statistical details of experiments**

| **Cannabinoid CB1 receptor in dorsal telencephalic glutamatergic neurons drives overconsumption of palatable food in obesity** | | | | |
| --- | --- | --- | --- | --- |
| **Figure number** | **Statistical analysis** | **Factor name** | **Statistic value** | **P-value** |
| Fig. 1a | Repeated measures  ANOVA | **Body weight (HFD)** |  |  |
|  |  | Time  Genotype  Time x Genotype | *F* (12,252) = 129.7 | *P* < 0.0001  *P* < 0.0001  *P* < 0.0001 |
|  |  |  | *F* (1,21) = 22.69  *F* (12,252) = 20.27 |  |
| Fig. 1b | Repeated measures  ANOVA | **Food intake (HFD)** |  |  |
|  |  | Time | *F* (11,231) = 3.132 | *P* < 0.001 |
|  |  | Genotype | *F* (1,21) = 12.06 | *P* < 0.01 |
|  |  | Time x Genotype | *F* (11,231) = 1.332 | n.s. |
| Fig. 1b | ANCOVA | **Food intake (HFD)**  Genotype  Body weight (covariate) | *F* (1,272) = 25.996  *F* (1,272) = 5.188 | *P* < 0.001  *P* < 0.05 |
| Fig. 1c | Repeated measures  ANOVA  Unpaired t-test | **Glucose Tolerance test** |  |  |
|  |  | Time | *F* (4,84) = 167 | *P* < 0.0001 |
|  |  | Genotype | *F* (1,21) = 6.713 | *P* < 0.05 |
|  |  | Time x Genotype  AUC | *F* (4,84) = 1.442  *t_(21)_*= 2.709 | n.s.  *P* < 0.05 |
| Fig. 1d | Repeated measures  ANOVA | **Insulin Tolerance test** |  |  |
|  |  | Time | *F* (4,80) = 89.05 | *P* < 0.0001 |
|  |  | Genotype | *F* (1,20) = 18.85 | *P* < 0.001 |
|  |  | Time x Genotype | *F* (4,80) = 5.547 | *P* < 0.001 |
| Fig. 1e | Repeated measures  ANOVA | **Body weight (pair-feeding test)**  Time  Genotype  Time x Genotype | *F* (12,180) = 134.3  *F* (1,15) = 1.827  *F* (12,180) = 5.008 | *P* < 0.0001  n.s.  *P* < 0.0001 |
| Fig. 1f | Repeated measures  ANOVA | **Energy expenditure**  Time  Genotype  Time x Genotype | *F* (192,2688) = 6.629  *F* (1,14) = 0.961  *F* (192,2688) = 0.959 | *P* < 0.0001  n.s.  n.s. |
| Fig. 1f | ANCOVA | **Energy expenditure**  Genotype  Body weight (covariate) | *F* (1,13) = 0.772  *F* (1,13) = 4.211 | n.s.  n.s. |
| Fig. 1g | Repeated measures  ANOVA | **Respiratory exchange ratio (RER)**  Time  Genotype  Time x Genotype | *F* (192,2688) = 5.511  *F* (1,14) = 0.054  *F* (192,2688) = 1.082 | *P* < 0.0001  n.s.  n.s. |
| Fig. 1h | Repeated measures  ANOVA | **Ambulatory activity**  Time  Genotype  Time x Genotype | *F* (192,2688) = 5.183  *F* (1,214) = 0.929  *F* (192,2688) = 1.046 | *P* < 0.0001  n.s.  n.s. |
| Fig. 2a | Repeated measures  ANOVA | **Body weight (LFD)** |  |  |
|  |  | Time | *F* (8,160) = 176.6 | *P* < 0.0001 |
|  |  | Genotype | *F* (1,20) = 0.3038 | n.s. |
|  |  | Time x Genotype | *F* (8,160) = 6.425 | *P* < 0.0001 |
| **Cannabinoid CB1 receptor in dorsal telencephalic glutamatergic neurons drives overconsumption of palatable food in obesity** | | | | |
| **Figure number** | **Statistical analysis** | **Factor name** | **Statistic value** | **P-value** |
| Fig. 2b | Repeated measures  ANOVA | **Food intake (LFD)** |  |  |
|  |  | Time | *F* (7,140) = 26.77 | *P* < 0.0001 |
|  |  | Genotype | *F* (1,20) = 1.082 | n.s. |
|  |  | Time x Genotype | *F* (7,140) = 2.421 | *P* < 0.05 |
| Fig. 2c | Repeated measures  ANOVA | **Body weight (Chocolate)** |  |  |
|  |  | Time | *F* (8,144) = 185.7 | *P* < 0.0001 |
|  |  | Genotype | *F* (1,18) = 4.53 | *P* < 0.05 |
|  |  | Time x Genotype | *F* (8,144) = 22.73 | *P* < 0.0001 |
| Fig. 2d | Repeated measures  ANOVA | **Food intake (Chocolate)** |  |  |
|  |  | Time | *F* (7,133) = 8.061 | *P* < 0.0001 |
|  |  | Genotype | *F* (1,19) = 3.751 | *P* = 0.0678 |
|  |  | Time x Genotype | *F* (7,133) = 0.4203 | n.s. |
| Fig. 2d | ANCOVA | **Food intake (Chocolate)**  Genotype  Body weight (covariate) | *F* (1,157) = 8.575  *F* (1,157) = 12.97 | *P* < 0.01  *P* < 0.001 |
| Fig. 2e | Repeated measures  ANOVA | **Body weight (Free-choice)** |  |  |
|  |  | Time | *F* (8,232) = 218.2 | *P* < 0.0001 |
|  |  | Genotype | *F* (1,29) = 18.02 | *P* < 0.001 |
|  |  | Time x Genotype | *F* (8,232) = 13.34 | *P* < 0.0001 |
| Fig. 2f | Repeated measures  ANOVA | **Food intake (HFD, free choice)** |  |  |
|  |  | Time  Genotype  Time x Genotype | *F* (7,112) = 17.91 | *P* < 0.001  *P* < 0.05  n.s. |
|  |  |  | *F* (1,16) = 4.667  *F* (7,112) = 1.329 |  |
| Fig. 2f | ANCOVA | **Food intake (HFD, free choice)**  Genotype  Body weight (covariate) | *F* (1,226) = 5.269  *F* (1,226) = 23.98 | *P* < 0.05  *P* < 0.001 |
| Fig. 2f | Repeated measures  ANOVA | **Food intake (LFD, free choice)** |  |  |
|  |  | Time | *F* (7,112) = 2.265 | *P* < 0.05 |
|  |  | Genotype | *F* (1,16) = 6.254 | *P* < 0.05 |
|  |  | Time x Genotype | *F* (7,112) = 0.594 | n.s. |
|  |  | **Progressive ratio 1 (PR1)** |  |  |
|  |  | Diet | *F* (1,75) = 1.96 | n.s. |
|  |  | Genotype | *F* (1,75) = 2.09 | n.s. |
|  |  | Diet x Genotype | *F* (1,75) = 3.29 | n.s. |
|  |  | **Progressive ratio 2 (PR2)** |  |  |
|  |  | Diet | *F* (1,75) = 4.39 | *P* < 0.05 |
|  |  | Genotype | *F* (1,75) = 3.63 | n.s. |
|  |  | Diet x Genotype | *F* (1,75) = 2.66 | n.s. |
| Fig. 3b | Two-Way  ANOVA | **Progressive ratio 3 (PR3)** |  |  |
|  |  | Diet | *F* (1,75) = 7.03 | *P* < 0.01 |
|  |  | Genotype | *F* (1,75) = 12.55 | *P* < 0.001 |
|  |  | Diet x Genotype | *F* (1,75) = 1.86 | n.s. |
|  |  |  |  |  |
|  | | | | |
| **Cannabinoid CB1 receptor in dorsal telencephalic glutamatergic neurons drives overconsumption of palatable food in obesity** | | | | |
| **Figure number** | **Statistical analysis** | **Factor name** | **Statistic value** | **P-value** |
|  |  | **Progressive ratio 4 (PR4)** |  |  |
|  |  | Diet | *F* (1,75) = 1.88 | n.s. |
|  |  | Genotype | *F* (1,75) = 1.55 | n.s. |
|  |  | Diet x Genotype | *F* (1,75) = 1.90 | n.s. |
|  |  | **Progressive ratio 5 (PR5)** |  |  |
|  |  | Diet | *F* (1,75) = 0.57 | n.s. |
|  |  | Genotype | *F* (1,75) = 0.04 | n.s. |
|  |  | Diet x Genotype | *F* (1,75) = 2.24 | n.s. |
|  |  | **Progressive ratio 6 (PR6)** |  |  |
|  |  | Diet | *F* (1,75) = 0.92 | n.s. |
|  |  | Genotype | *F* (1,75) = 3.02 | n.s. |
| Fig. 3b | Two-Way  ANOVA | Diet x Genotype | *F* (1,75) = 2.73 | n.s. |
|  |  | **Progressive ratio 7 (PR7)** |  |  |
|  |  | Diet | *F* (1,75) = 0.92 | n.s. |
|  |  | Genotype | *F* (1,75) = 3.02 | n.s. |
|  |  | Diet x Genotype | *F* (1,75) = 2.73 | *P* < 0.05 |
|  |  | **Progressive ratio 8 (PR8)** |  |  |
|  |  | Diet | *F* (1,75) = 1.02 | n.s. |
|  |  | Genotype | *F* (1,75) = 8.76 | *P* < 0.01 |
|  |  | Diet x Genotype | *F* (1,75) = 8.35 | *P* < 0.01 |
|  |  | **Progressive ratio 9 (PR9)** |  |  |
|  |  | Diet | *F* (1,75) = 1.56 | n.s. |
|  |  | Genotype | *F* (1,75) = 12.46 | *P* < 0.001 |
|  |  | Diet x Genotype | *F* (1,75) = 6.20 | *P* < 0.05 |
| Fig. 4a | Repeated measures  ANOVA | **Habituation odor test (HFD)** |  |  |
|  |  | Odor | *F* (2,38) = 63.71 | *P* < 0.0001 |
|  |  | Genotype | *F* (2,19) = 18.33 | *P* < 0.0001 |
|  |  | Odor x Genotype | *F* (4,38) = 8.437 | *P* < 0.0001 |
| Fig. 4a | Repeated measures  ANOVA | **Habituation odor test (chocolate)** |  |  |
|  |  | Odor | *F* (2,38) = 43.08 | *P* < 0.0001 |
|  |  | Genotype | *F* (1,19) = 7.325 | *P* < 0.05 |
|  |  | Odor x Genotype | *F* (2,38) = 8.512 | *P* < 0.001 |
| Fig. 4a | Repeated measures  ANOVA | **Habituation odor test (free choice)** |  |  |
|  |  | Odor | *F* (2,60) = 52.6 | *P* < 0.0001 |
|  |  | Genotype | *F* (1,30) = 1.52 | n.s. |
|  |  | Odor x Genotype | *F* (2,60) = 4.331 | *P* < 0.05 |
| Fig. 4b | Unpaired t-test | **Buried food test** |  |  |
|  |  | Body weight | *t_(28)_*= 4.636 | *P* < 0.0001 |
|  |  | Latency | *t_(28)_*= 2.224 | *P* < 0.05 |
|  |  |  |  |  |
| Fig. 5b | Two-way  ANOVA | **CB1 mRNA expression (mouse + transgene CB1 primers)** |  |  |
|  |  | AAV | *F* (1,14) = 5.074 | *P* < 0.05 |
|  |  | Genotype | *F* (1,14) = 0.0002 | n.s. |
|  |  | AAV x Genotype | *F* (1,14) = 4.34 | *P* = 0.056 |
| **Cannabinoid CB1 receptor in dorsal telencephalic glutamatergic neurons drives overconsumption of palatable food in obesity** | | | | |
| **Figure number** | **Statistical analysis** | **Factor name** | **Statistic value** | **P-value** |
| Fig. 5b | Two-way  ANOVA | **CB1 mRNA expression (mouse CB1 primers)** |  |  |
|  |  | AAV | *F* (1,14) = 0.01068 | n.s. |
|  |  | Genotype | *F* (1,14) = 31.58 | *P* < 0.0001 |
|  |  | AAV x Genotype | *F* (1,14) = 0.05249 | n.s. |
| Fig. 5d | Two-way  ANOVA | **Cumulative weight gain** |  |  |
|  |  | AAV  Genotype  AAV x Genotype | *F* (1,27) = 4.034 | P=0.0547  *P* < 0.01  *P* < 0.05 |
|  |  |  | *F* (1,27) = 8.452  *F* (1,27) = 4.262 |  |
| Fig. 5e | Two-way  ANOVA | **Food intake** |  |  |
|  |  | AAV | *F* (1,28) = 4.296 | *P* < 0.05 |
|  |  | Genotype | *F* (1,28) = 0.006 | n.s. |
|  |  | AAV x Genotype | *F* (1,28) = 7.684 | *P* < 0.01 |
| Fig. 5f | Two-way  ANOVA | **Habituation odor test** |  |  |
|  |  | AAV | *F* (1,24) = 0.034 | n.s. |
|  |  | Genotype | *F* (1,24) = 8.432 | *P* < 0.01 |
|  |  | AAV x Genotype | *F* (1,24) = 2.45 | n.s. |
| Fig. 5g | Two-way  ANOVA | **Glucose tolerance test (AUC)** |  |  |
|  |  | AAV | *F* (1,22) = 0.274 | n.s. |
|  |  | Genotype | *F* (1,22) = 3.969 | *P* = 0.0589 |
|  |  | AAV x Genotype | *F* (1,22) = 4.466 | *P* < 0.05 |
| Suppl. Fig. 2 | Unpaired t-test | **Food preference**  HFD (g) /total food (g) | *t_(29)_*= 2.506 | *P* < 0.05 |
| Suppl. Fig. 3a | Repeated measures  ANOVA | **Number of reinforcers in 1h**  **FR1**  Diet  Genotype  Diet x Genotype  Time  Time x Diet  Time x Genotype  Time x Diet x Genotype  **FR5**  Diet  Genotype  Diet x Genotype  Time  Time x Genotype  Time x Diet  Time x Diet x Genotype | *F* (1,75) = 0.25  *F* (1,75) = 0.12  *F* (1,75) = 0.02  *F* (4,300) = 6.71  *F* (4,300) = 0.68  *F* (4,300) = 3.82  *F* (4,300) = 0.56  *F* (1,75) = 0.86  *F* (1,75) = 0.67  *F* (1,75) = 1.46  *F* (4,300) = 4.83  *F* (4,300) = 0.15  *F* (4,300) = 1.13  *F* (4,300) = 0.82 | n.s.  n.s.  n.s.  *P* <0.001  n.s.  *P* <0.01  n.s.  n.s.  n.s.  n.s.  *P* <0.001  n.s.  n.s.  n.s. |
| Suppl. Fig. 3b  Suppl Fig. 3b | Repeated measures  ANOVA  Repeated measures  ANOVA | **Number of lever-presses in 1h**  **FR1**  Diet  Genotype  Diet x Genotype  Time  Time x Genotype  Time x Diet  Time x Diet x Genotype  **FR5**  Diet  Genotype  Diet x Genotype  Time  Time x Genotype  Time x Diet  Time x Diet x Genotype | *F* (1,75) = 0.03  *F* (1,75) = 0.22  *F* (1,75) = 0.01  *F* (4,300) = 2.95  *F* (4,300) = 3.46  *F* (4,300) = 0.53  *F* (4,300) = 0.74  *F* (1,75) = 0.63  *F* (1,75) = 1.07  *F* (1,75) = 1.22  *F* (4,300) = 5.21  *F* (4,300) = 0.14  *F* (4,300) = 0.97  *F* (4,300) = 0.68 | n.s.  n.s.  n.s.  *P* <0.05  *P* <0.01  n.s.  n.s.  n.s.  n.s.  n.s.  *P* <0.001  n.s.  n.s.  n.s. |
| Suppl Fig. 3c | Repeated measures  ANOVA | **Body weight (g)**  Diet  Genotype  Diet x Genotype  Time  Time x Genotype  Time x Diet  Time x Diet x Genotype | *F* (1,75) = 0.14  *F* (1,75) = 3.43  *F* (1,75) = 3.45  *F* (10,750) =438.947  *F* (10,750) =1.50  *F* (10,750) =4.35  *F* (10,750) =2.57 | n.s.  n.s.  n.s.  *P* <0.001  *P* <0.001  n.s.  *P* <0.01 |
| Suppl Fig. 3d | Repeated measures  ANOVA | **Food intake (calories/day)**  Diet  Genotype  Diet x Genotype  Time  Time x Genotype  Time x Diet  Time x Diet x Genotype | *F* (1,75) = 45.09  *F* (1,75) = 2.88  *F* (1,75) =2.17  *F* (10,750) = 22.06  *F* (10,750) = 2.51  *F* (10,750) = 29.53  *F* (10,750) = 0.33 | n.s.  n.s.  n.s.  *P* <0.001  *P* <0.01  *P* <0.001  n.s. |
| Suppl. Fig. 4b  Suppl. Fig. 4c  Suppl. Fig. 4d | One-way ANOVA  One-way ANOVA  Two-way  ANOVA | **CB1 receptor expression (Western-blot)**  **CB1 mRNA expression (qPCR)**  **CB1 receptor expression (Synaptosomes)**  Fractions  Genotype  Fractions x Genotype | *F* (3,14) = 23.54  *F* (2,10) = 21.45 | *P* < 0.0001  *P* < 0.001  *P* < 0.05  *P* < 0.01  n.s. |
|  |  |  | *F* (1,12) = 5.258  *F* (1,12) = 17.55  *F* (1,12) = 1.728 |  |
| Suppl. Fig. 5a | Repeated measure  ANOVA | **Habituation odor test (LFD-fed mice)** |  |  |
|  |  | Odor | *F* (2,40) = 18.17 | *P* < 0.0001 |
|  |  | Genotype  Odor x Genotype | *F* (1,20) = 0.7322  *F* (2,40) = 0.6424 | n.s.  n.s. |
|  |  |  |  |  |
| Suppl. Fig. 5b | Unpaired t-test | **Habituation odor test (before BW changes)**  Body weight  Odor  Genotype  Odor x Genotype | *t_(35)_*= 0.886  *F* (2,66) = 46.77  *F* (1,33) = 1.106  *F* (2,66) = 0.529 | n.s.  *P* < 0.0001  n.s  n.s.. |
| Suppl. Fig. 5c | Pearson correlation | **Exploration time x Body weight** | *r_(33)_*= 0.5078 | *P* < 0.01 |
| Suppl. Fig. 5d | Repeated measure  ANOVA | **Habituation odor test (24 hours fasting)**  Odor  Genotype  Odor x Genotype | *F* (2,34) = 45.7  *F* (2,17) = 5.269  *F* (4,34) = 1.571 | *P* < 0.0001  *P* < 0.05  n.s. |
| **Cannabinoid CB1 receptor in dorsal telencephalic glutamatergic neurons drives overconsumption of palatable food in obesity** | | | | |
| **Figure number** | **Statistical analysis** | **Factor name** | **Statistic value** | **P-value** |
| Suppl. Fig. 5e | Unpaired t-test | **Buried food test (LFD)**  Latency | *t* (29)= 2.355 | *P* < 0.05 |
| Suppl. Fig. 7a | Repeated measures  ANOVA | **Body weight** |  |  |
|  |  | Time  AAV and Genotype  Time x AAV and Genotype | *F* (8,216) = 138.1 | *P* < 0.0001  *P* = 0.001  *P* < 0.0001 |
|  |  |  | *F* (3,27) = 7.29  *F* (24,216) = 3.275 |  |
| Suppl. Fig. 7b | Repeated measures  ANOVA | **Food intake** |  |  |
|  |  | Time | *F* (7,189) = 33.81 | *P* < 0.0001 |
|  |  | AAV and Genotype | *F* (3,27) = 6.843 | *P* < 0.01 |
|  |  | Time x AAV and Genotype | *F* (21,189) = 2 | *P* < 0.01 |
| Suppl. Fig. 7c | Repeated measures  ANOVA | **Habituation odor test** |  |  |
|  |  | Odor | *F* (2,72) =59.17 | *P* < 0.0001 |
|  |  | AAV and Genotype | *F* (3,72) = 3.347 | *P* < 0.05 |
|  |  | Odor x AAV and Genotype | *F* (6,72) = 3.46 | *P* <0.01 |
| Suppl. Fig. 7d | Repeated measures  ANOVA | **Glucose tolerance test** |  |  |
|  |  | Time | *F* (4,88) = 211 | *P* < 0.0001 |
|  |  | AAV and Genotype  Time x AAV and Genotype | *F* (3,22) = 3.188  *F* (12,88) = 0.871 | *P* < 0.05  n.s. |
