# Supplementary figures and images for: Cannabinoid CB1 receptor in dorsal telencephalic glutamatergic neurons drives overconsumption of palatable food and obesity
Source: Neuropsychopharmacology. 2021 Feb 8;46(5):982–91. doi: 10.1038/s41386-021-00957-z (PMC8105345; doi:10.1038/s41386-021-00957-z)

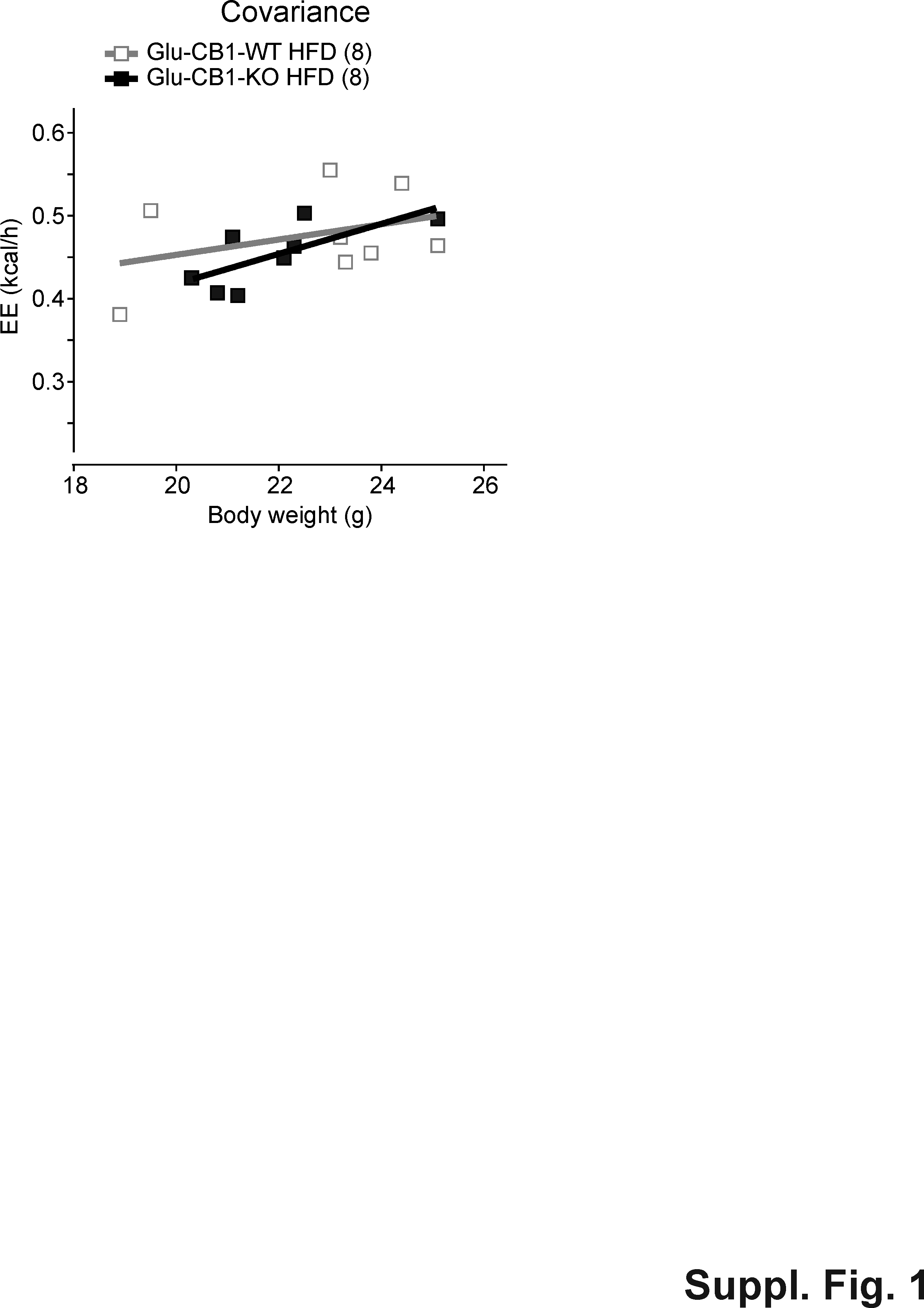

Supplement: Supplementary file 4 — Figure S1 [file 41386_2021_957_MOESM4_ESM.tif]

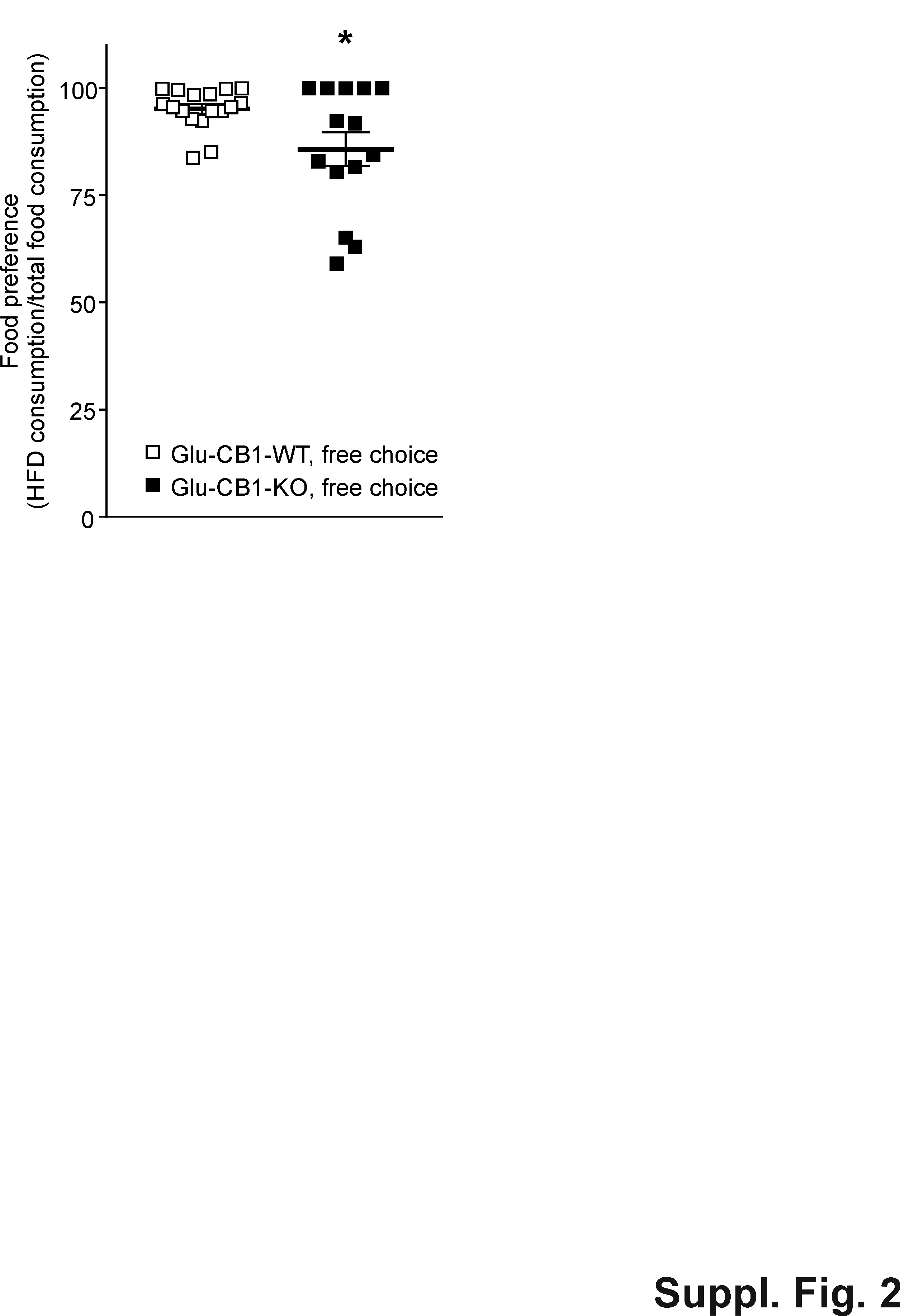

Supplement: Supplementary file 5 — Figure S2 [file 41386_2021_957_MOESM5_ESM.tif]

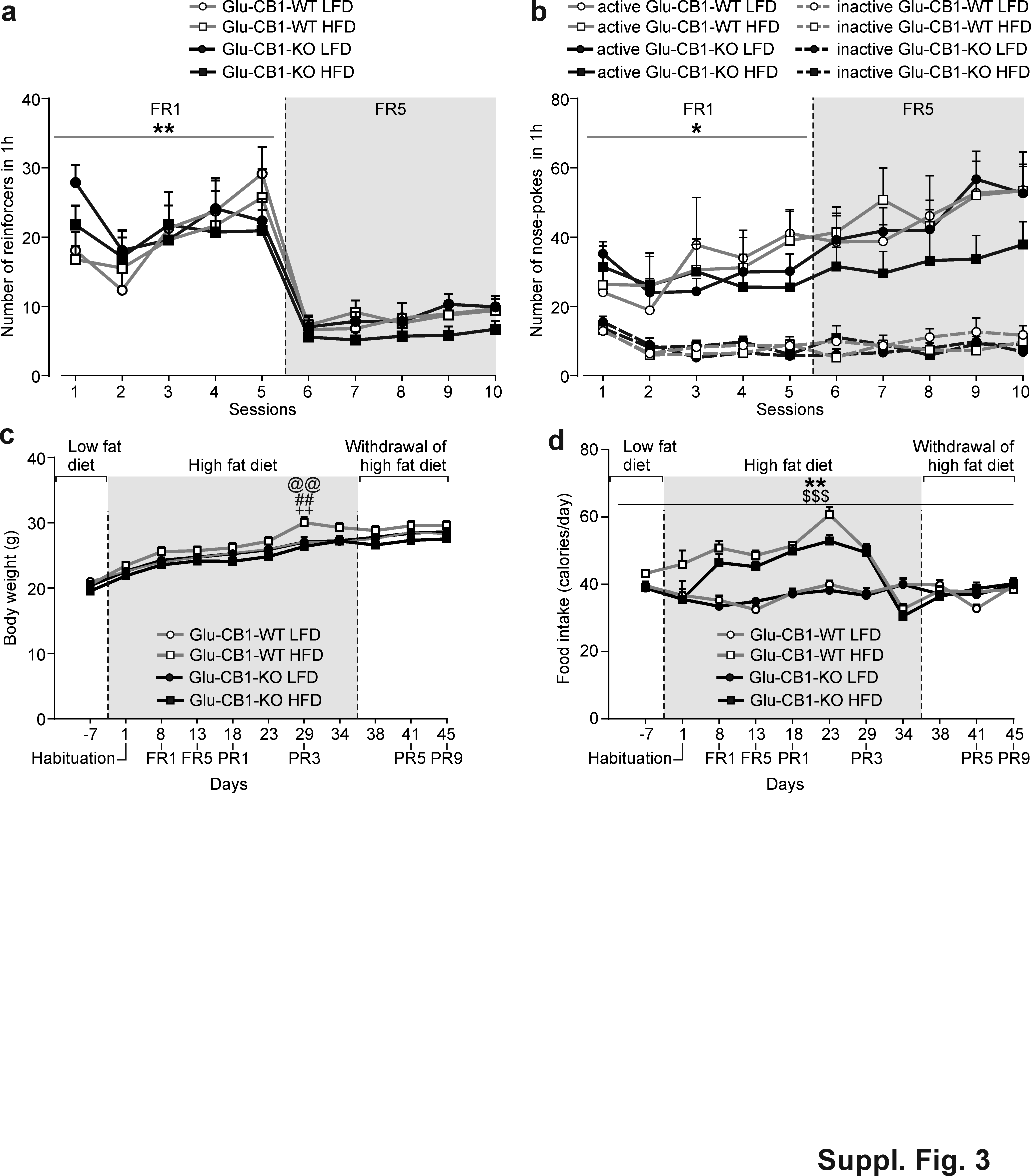

Supplement: Supplementary file 6 — Figure S3 [file 41386_2021_957_MOESM6_ESM.tif]

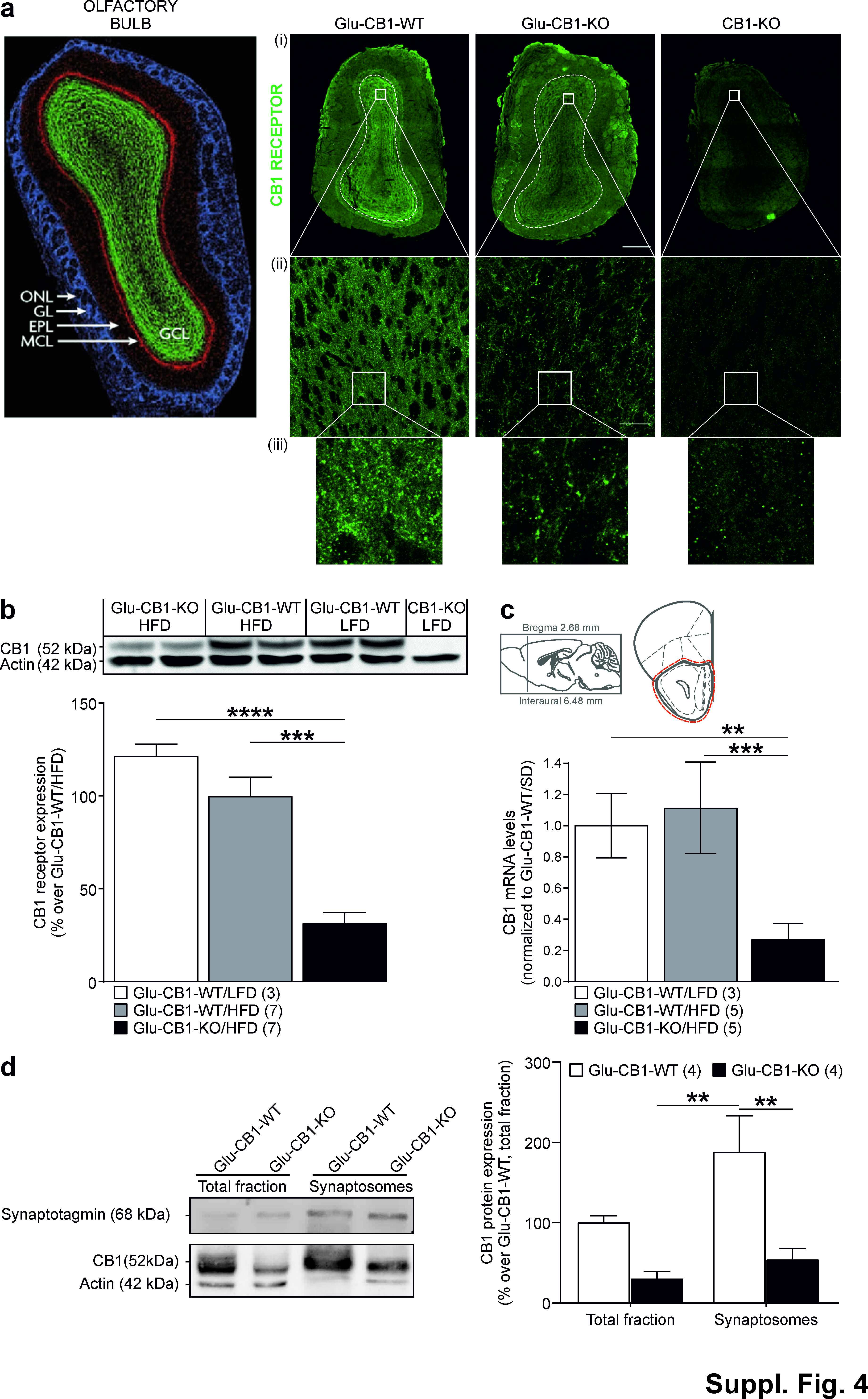

Supplement: Supplementary file 7 — Figure S4 [file 41386_2021_957_MOESM7_ESM.tif]

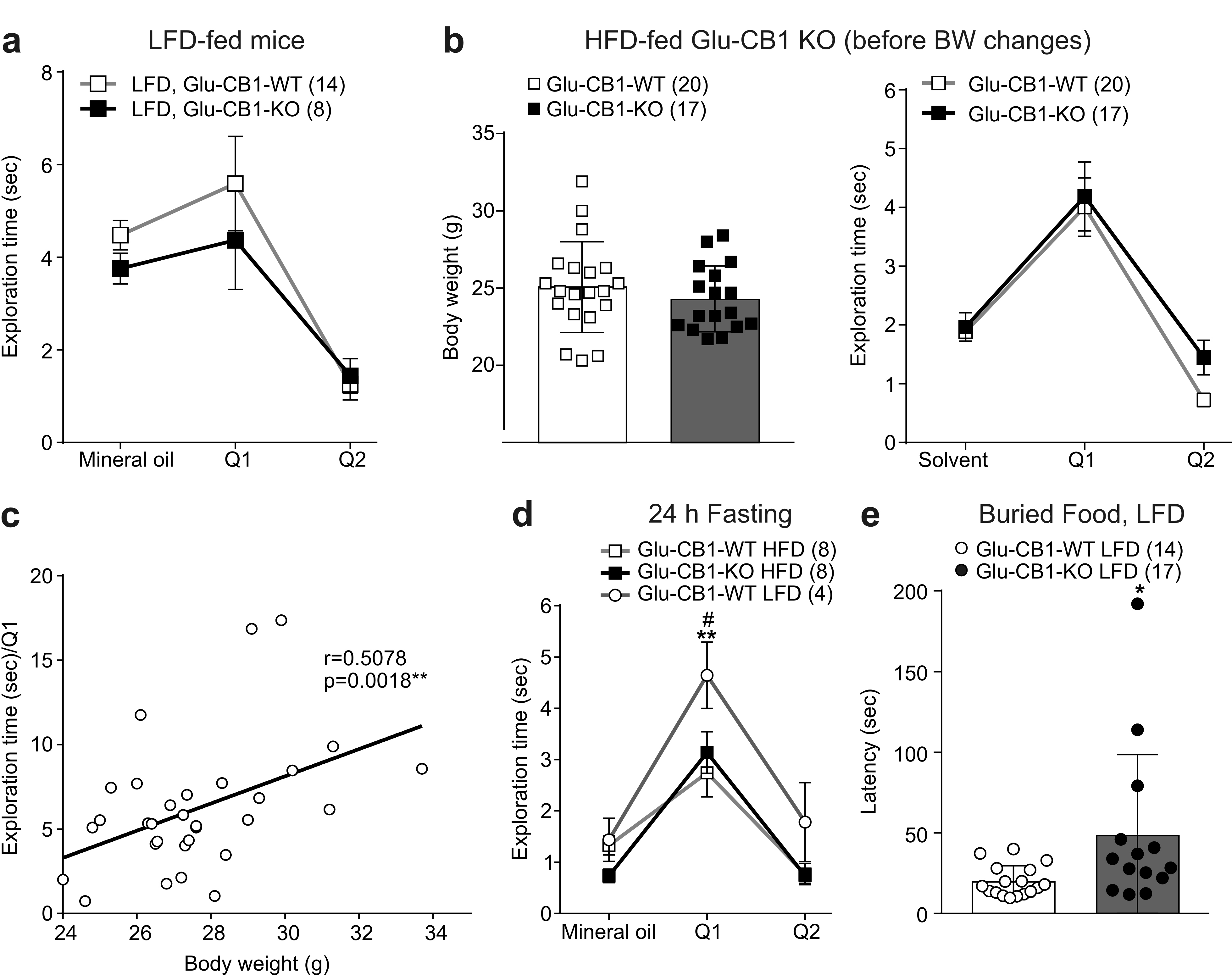

Supplement: Supplementary file 8 — Figure S5 [file 41386_2021_957_MOESM8_ESM.tif]

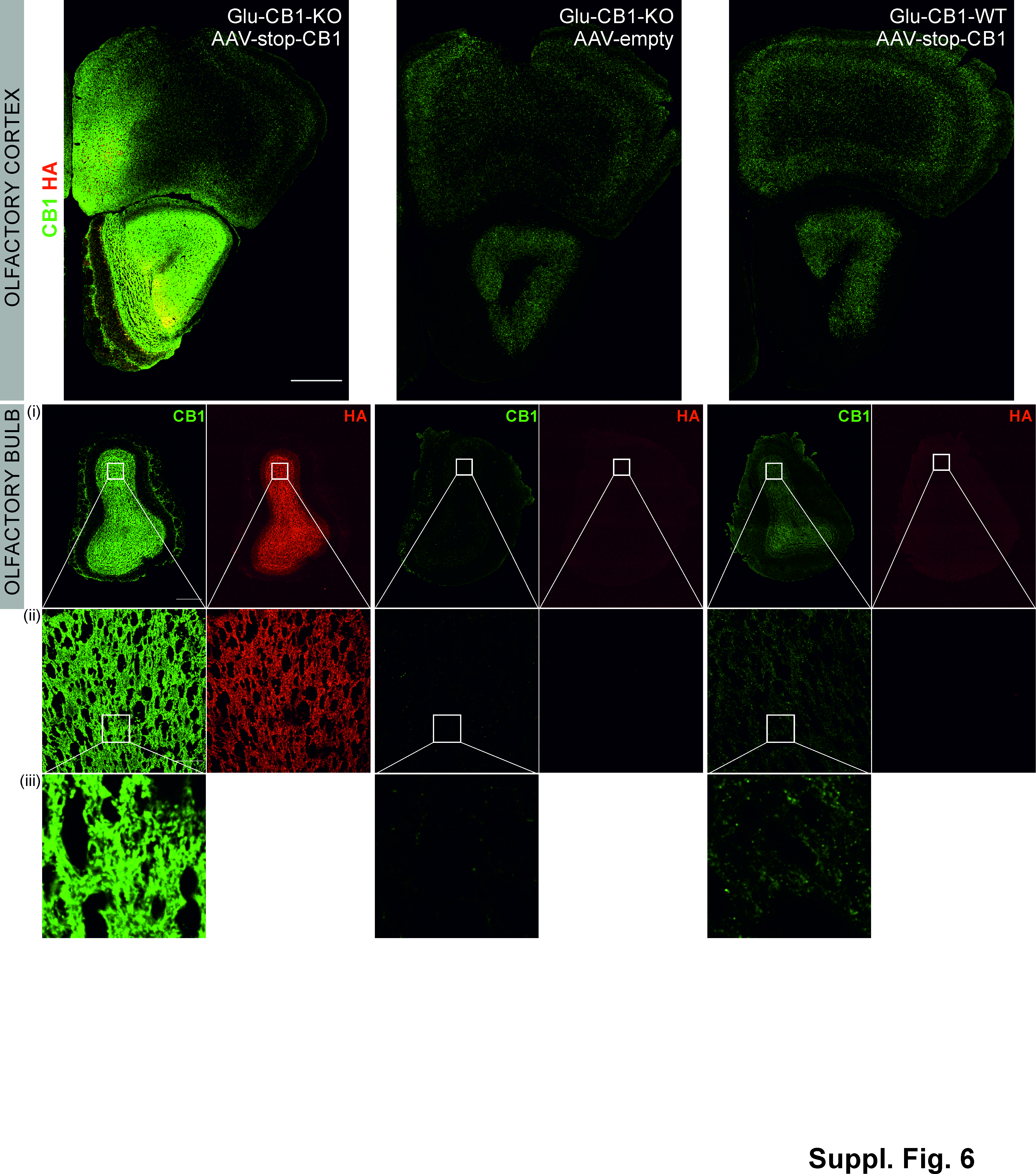

Supplement: Supplementary file 9 — Figure S6 [file 41386_2021_957_MOESM9_ESM.tif]

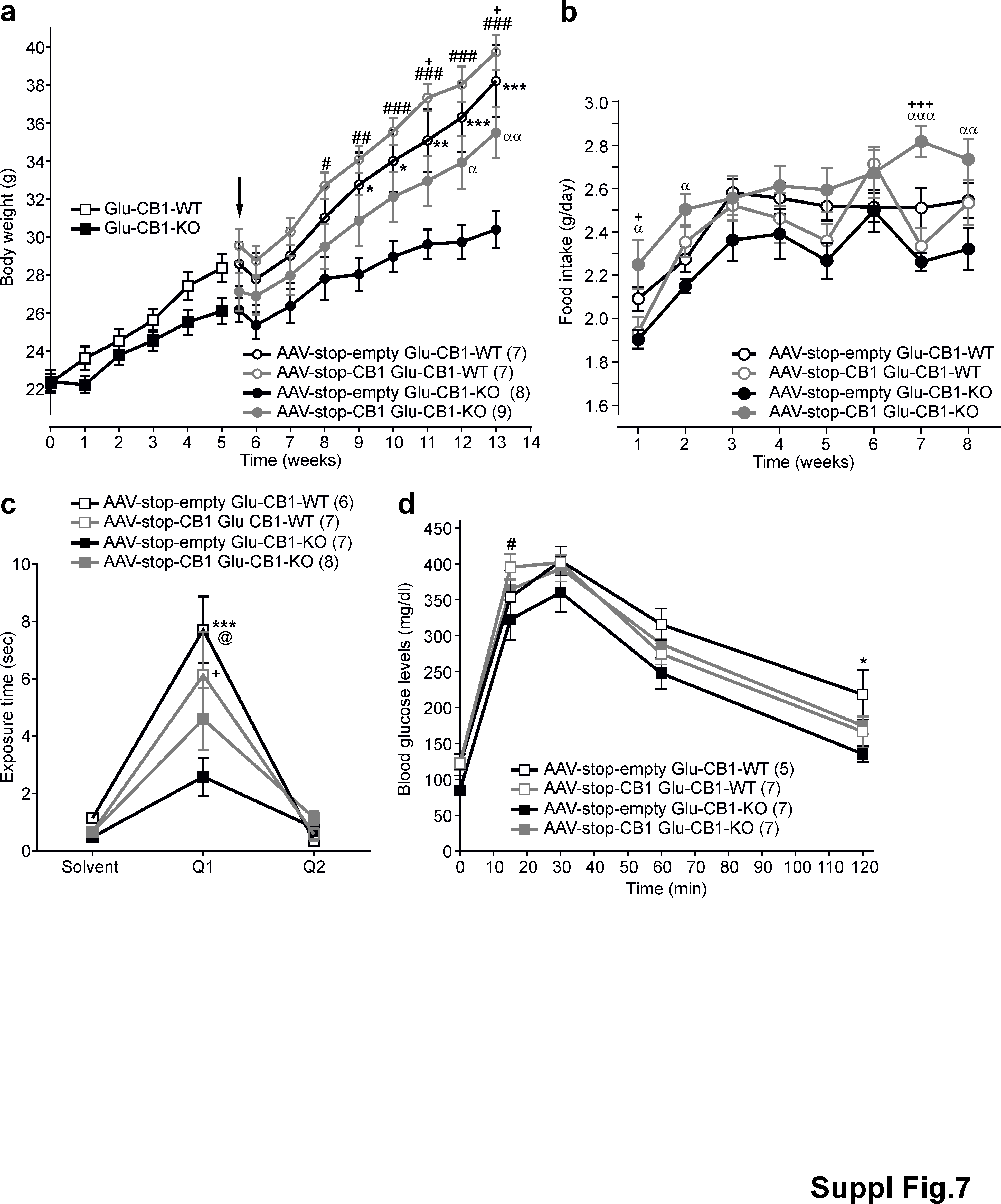

Supplement: Supplementary file 10 — Figure S7 [file 41386_2021_957_MOESM10_ESM.tif]
